# Mitochondrial dysfunction in pancreatic acinar cells: mechanisms and therapeutic strategies in acute pancreatitis

**DOI:** 10.3389/fimmu.2024.1503087

**Published:** 2024-12-24

**Authors:** Fan Chen, Kedong Xu, Yimin Han, Jiachun Ding, Jiaqiang Ren, Yaochun Wang, Zhenhua Ma, Fang Cao

**Affiliations:** ^1^ Department of Hepatobiliary Surgery, The First Affiliated Hospital of Xi’an Jiaotong University, Xi’an, China; ^2^ Pancreatic Disease Center of Xi’an Jiaotong University, Xi’an, China; ^3^ Center for Translational Medicine, The First Affiliated Hospital of Xi’an Jiaotong University, Xi’an, China

**Keywords:** acute pancreatitis, mitochondrial, calcium overload, mitochondrial permeability transition pore, regulated cell death

## Abstract

Acute pancreatitis (AP) is an inflammatory disease of the pancreas and a complex process involving multiple factors, with mitochondrial damage playing a crucial role. Mitochondrial dysfunction is now considered a key driver in the development of AP. This dysfunction often presents as increased oxidative stress, altered membrane potential and permeability, and mitochondrial DNA damage and mutations. Under stress conditions, mitochondrial dynamics and mitochondrial ROS production increase, leading to decreased mitochondrial membrane potential, imbalanced calcium homeostasis, and activation of the mitochondrial permeability transition pore. The release of mitochondrial DNA (mtDNA), recognized as damage-associated molecular patterns, can activate the cGAS-STING1 and NF-κB pathway and induce pro-inflammatory factor expression. Additionally, mtDNA can activate inflammasomes, leading to interleukin release and subsequent tissue damage and inflammation. This review summarizes the relationship between mitochondria and AP and explores mitochondrial protective strategies in the diagnosis and treatment of this disease. Future research on the treatment of acute pancreatitis can benefit from exploring promising avenues such as antioxidants, mitochondrial inhibitors, and new therapies that target mitochondrial dysfunction.

## 1 Introduction

Acute pancreatitis (AP) is a common condition in the digestive system, characterized by the abnormal activation of pancreatic enzymes that can lead to inflammation in the pancreas and surrounding organs (1). The annual incidence rate of AP varies from 4.9 to 73.4 per 100,000 individuals, depending on factors such as regional differences, variations in diagnostic criteria, and population demographics, with a rising trend (2, 3). For instance, higher incidence rates are often reported in Western countries due to lifestyle factors such as alcohol consumption and gallstone prevalence, whereas lower rates are observed in regions with different dietary and health risk profiles (4). Additionally, improvements in diagnostic techniques and greater awareness of the disease in recent decades have contributed to the rising trend in AP incidence globally (3, 5). While most patients experience mild symptoms, 20% to 30% may progress to severe acute pancreatitis (SAP), which often involves organ dysfunction. In SAP cases, the mortality rate can be as high as 20% to 40%, posing a significant threat to individuals’ lives and well-being (6). Pancreatic damage typically originates in acinar cells, the primary cell type of the exocrine pancreas, which leads to inflammation as a secondary process. AP is commonly associated with elevated levels of digestive enzymes in the blood, such as amylase and lipase, though these are not strict requirements for diagnosis. Premature activation of digestive enzymes (such as the conversion of trypsinogen to trypsin), formation of large vacuoles within acinar cells, and activation of inflammatory mediators are also key features of the disease. While hyperamylasemia is frequently observed, lipase elevation is considered more specific for diagnosing AP (7, 8). This includes the key transcription factor nuclear factor kappa B (NF-κB), which triggers the infiltration of inflammatory cells in the pancreas and systemic inflammatory response, leading to acinar cell death through apoptosis and necrosis (9). A significant body of research has explored the signaling pathways involved in these pathological processes, elucidating the structure of many molecules that mediate inflammatory responses (e.g., NF-κB, cytokines/chemokines, adhesion molecules, and novel protein kinase C subtypes) and cell death responses (e.g., cysteine enzymes) (10–12). However, other areas of AP research, such as the impact of damage to intracellular organelles like mitochondria, remain incompletely understood, warranting further investigation.

Recent studies have highlighted the importance of intracellular organelles, particularly mitochondria, in the progression of AP. Mitochondrial dysfunction, characterized by impaired ATP synthesis and increased production of reactive oxygen species (ROS), has been implicated in exacerbating pancreatic inflammation and cell death.

Activation of pattern recognition receptors (PRRs) in both immune and non-immune cells often trigger inflammation. These receptors can be activated not only by viruses and bacteria, known as microbe-associated molecular patterns or pathogen-associated molecular patterns, but also by endogenous molecules known as damage-associated molecular patterns (DAMPs) (13). Under normal conditions, DAMPs such as nucleic acids, ATP, and calreticulin are unable to stimulate PRRs due to limited access to subcellular regions where PRRs are located. During inflammation, however, changes in membrane permeability allow these molecules to activate PRRs and drive the inflammatory process (14). Various mitochondrial elements and metabolites can act as DAMPs, potentially triggering inflammatory responses upon their release into the cytosol or the external environment. Research has demonstrated that during AP, changes in membrane permeability of acinar cells and organelles result in mitochondrial DNA (mtDNA) being released and the NLRP3 inflammasome becoming activated, thereby driving inflammation (15).

Research on the pathophysiological mechanisms of AP has advanced, but the precise mechanism of cell damage during inflammation remains unclear, hindering effective clinical treatments. Mitochondria, as the primary energy producers in cells, play a crucial role in cellular homeostasis and survival. Dysfunctional mitochondria contribute to the pathogenesis of AP by disrupting ATP synthesis, increasing ROS production, and altering calcium homeostasis. Studies indicate that intracellular calcium overload in AP leads to mitochondrial dysfunction, disrupting ATP synthesis and causing acinar cell damage and necrosis, exacerbating inflammation (16–18). The severity of AP correlates with necrosis extent, with mitochondria influencing autophagy and apoptosis regulation. This review explores mitochondria’s physiological role in pancreatic function, emphasizing their dysfunction’s role in AP through mechanisms such as calcium overload, ATP depletion, oxidative stress, and membrane permeability changes. Additionally, it also discusses mitochondria-related signaling pathways in AP and proposes potential therapeutic strategies targeting mitochondrial dysfunction for improved AP diagnosis and treatment.

## 2 Mitochondria: structure, origin, and the role in cellular function and pathophysiology

**Figure 1:**
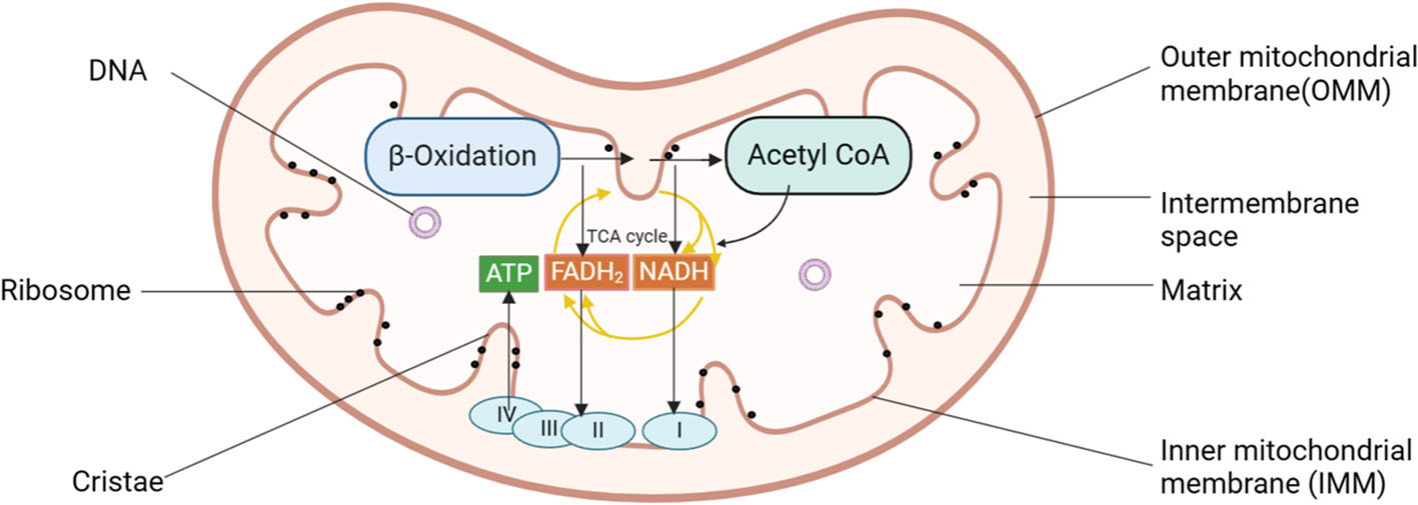
Diagram of mitochondrial structure pattern of pancreatic acinar cells. Mitochondria can be categorized into four distinct regions: the OMM, the mitochondrial intermembrane space, the IMM, and the mitochondrial matrix. Mitochondria serve as the primary sites for oxidative phosphorylation and ATP production within cells, fueling cellular activities. I, complex I, NADH: ubiquinone oxidoreductase; II, complex II, succinate: ubiquinone oxidoreductase; III, complex III, ubiquinol: cytochrome c oxidoreductase; IV, cytochrome c oxidase; ATP, adenosine triphosphate; IMM, inner mitochondrial membrane; OMM, outer mitochondrial membrane.

Mitochondria are crucial organelles in eukaryotic cells, often recognized as the cell’s powerhouse due to their critical role in energy production. Structurally, they can be categorized into four distinct regions: the outer mitochondrial membrane (OMM), the mitochondrial intermembrane space, the inner mitochondrial membrane (IMM), and the mitochondrial matrix, each contributing to the organelle’s unique and complex functionality (19). These structures are integral to the mitochondria’s ability to support cellular functions, particularly in energy production through oxidative phosphorylation. Mitochondria are the primary sites for oxidative phosphorylation and ATP production within cells, providing energy for cellular activities (20). The tricarboxylic acid (TCA) cycle breaks down the carbon substrate of acetyl-CoA, derived from pyruvate, amino acids, and fatty acids, generating carbon dioxide and reducing NAD^+^ to NADH and FAD^2+^ to FADH_2_ (21) (see **Figure 1**). These molecules then serve as substrates for the respiratory chain, driving ATP production through oxidative phosphorylation. The activity of the rate-limiting enzyme in the TCA cycle is dependent on Ca^2+^, which aids mitochondria in adjusting to heightened cellular ATP demand.

Under normal conditions, Ca^2+^ accumulation in mitochondria remains stable. Ca^2+^ is released from the ER, triggering zymogen exocrine secretion and promoting ATP production in the mitochondria. However, the rise in cytoplasmic calcium ion concentration is brief as ATP-dependent calcium ion channels swiftly clear the cytoplasmic calcium ions. The sarcoplasmic/endoplasmic reticulum calcium ATPases (SERCAs) move calcium ions into the ER, while plasma membrane Ca^2+^ ATPases (PMCAs) transport calcium ions out of the cell. Prolonged Ca^2+^ influx can result in intracellular calcium overload, leading to mitochondrial membrane damage, alterations in mitochondrial membrane potential, and decreased ATP production (22, 23). This disruption is particularly detrimental in the context of AP, where calcium overload accelerates mitochondrial injury and contributes to acinar cell death.

Mitochondria are also essential in producing reactive oxygen species (ROS), which are by-products of ATP generation and have various intracellular effects. Mitochondrial ROS (mitoROS), including reactive ions and molecules, such as superoxide anion (O^2-^), hydrogen peroxide (H_2_O_2_), and hydroxyl radical (-OH), are produced by the electron transport chain in mitochondria. The generation of mitoROS primarily occurs in the mitochondrial respiratory chain during the electron transfer process. While these ROS participate in cell signaling and regulation under normal circumstances, excessive production or inadequate clearance can lead to cellular damage. Low ROS levels can promote cell proliferation. Kirova et al. discovered that mitoROS directly regulate cyclin-dependent kinase 2 (CDK2), targeting a specific conserved cysteine and disrupting the regulatory CDK-related phosphatase (KAP), thus influencing the cell cycle (24). However, elevated mitoROS levels have been linked to pathological conditions, such as pancreatitis, where excessive ROS production induces oxidative stress and triggers apoptosis pathways (25).

Moreover, mitochondria regulate intracellular calcium levels, and their strategic localization within the cell helps compartmentalize calcium signals. Disturbances in calcium homeostasis lead to mitochondrial calcium overload, which disrupts the mitochondrial membrane potential, decreases ATP production, and ultimately triggers acinar cell death through apoptosis and necrosis (26). Thus, the delicate balance between mitochondrial calcium regulation and ROS production is essential for cellular survival, and its disruption is a key driver of pancreatic cell injury in AP.

In pancreatic acinar cells, mitochondria are identified into three distinct groups: perigranular mitochondria at the granular and basolateral boundary, peripheral mitochondria in the basolateral zone near the plasma membrane, and perinuclear mitochondria surrounding the cell nucleus (27–29). These groups of mitochondria play specific roles in regulating intracellular Ca^2+^ homeostasis. Perigranular mitochondria prevent the diffusion of Ca^2+^ signals into the basal region, confining physiological Ca^2+^ signals to the apical region. Peripheral mitochondria supply ATP for Ca^2+^ pump-mediated uptake into the endoplasmic reticulum (ER) and are implicated in store-operated calcium influx. Perinuclear mitochondria act as a protective shield for the nucleus, protecting it from Ca^2+^ signal intrusion (30, 31). This spatial organization is essential for maintaining physiological Ca^2+^ dynamics in pancreatic cells, and its disruption is closely tied to mitochondrial dysfunction in AP.

One significant connection is seen in mitochondrial diseases such as Kearns-Sayre syndrome, where mitochondrial dysfunction directly links to recurrent episodes of pancreatitis, highlighting the impact of disrupted mitochondrial metabolism on pancreatic tissue (32). In AP, mitochondrial injury, particularly through calcium signaling dysregulation, leads to necrosis of pancreatic acinar cells. Calcium overload in mitochondria specifically contributes to necrotic injury, underscoring the importance of maintaining mitochondrial health to prevent acinar cell death (33). Central to this pathology is mitochondrial dysfunction, driving energy deficits and cellular necrosis. This is exacerbated in injured pancreatic acinar cells by impaired macroautophagy, hindering the critical process of clearing damaged mitochondria (34). Studies have emphasized that mitochondrial damage occurs early in the progression of AP, not only affecting the pancreas but also impacting organs like the lungs and jejunum, suggesting that mitochondrial dysfunction contributes to the systemic manifestations of the disease (35). In addition to mitochondrial dysfunction, disorders in calcium metabolism are crucial in promoting cell injury and necrosis in AP. The interplay between mitochondrial calcium overload and energy failure sets the stage for acinar cell death and tissue damage. Given the central role of calcium-mediated mitochondrial dysfunction in AP pathogenesis, therapeutic strategies targeting mitochondrial protection and calcium regulation have been proposed to mitigate disease severity (36). Emerging therapeutic approaches focusing on mitochondrial health offer new hope for AP treatment. Recent research suggests that the delivery of hypoxia-treated functional mitochondria to damaged pancreatic acinar cells by mesenchymal stem cells can alleviate metabolic dysfunction and reduce tissue injury (37). Additionally, mitochondria-targeted therapies, such as Kaempferol nanoparticles, have demonstrated potential in improving mitochondrial homeostasis and reducing inflammation in models of SAP (38).

The distribution of mitochondria within pancreatic ductal epithelial (PDE) cells is still not well understood. Electron microscopy studies in guinea pig PDE cells have shown that mitochondria are most densely packed in the cell’s apical portion (39). The functional importance of this localization remains currently unknown, but it could potentially support the energy requirements for ion secretion across the apical membrane of PDE cells.

## 3 Mitochondrial dysfunction in acute pancreatitis pathogenesis

### 3.1 Mitochondrial calcium overload

Calcium overload is a significant factor causing acute pancreatitis (AP) in pancreatic acinar cells. Calcium ions (Ca^2+^) serve as crucial second messengers in cells and act as essential cofactors for multiple digestive enzymes within acinar cells. Proper regulation of Ca^2+^ levels is vital for sustaining cell functions such as metabolism, proliferation, differentiation, apoptosis, and other cellular processes. The regulation of mitochondrial calcium homeostasis is primarily controlled by the mitochondrial calcium uniporter (MCU), which mediates the uptake of Ca^2+^ into the mitochondria. During pathological conditions such as AP, MCU becomes hyperactive, leading to excessive Ca^2+^ accumulation in the mitochondrial matrix, which overwhelms the mitochondria’s buffering capacity. This calcium overload promotes mitochondrial membrane depolarization, impairs ATP synthesis, and triggers cell death through necrotic pathways. A study by M. Chvanov et al. found that knocking out MCU influences mitochondrial Ca^2+^ dynamics but does not reduce the severity of experimentally induced AP. This suggests that while MCU facilitates mitochondrial Ca^2+^ uptake, its role in AP pathogenesis may involve compensatory mechanisms or other factors that mitigate its impact (40). Conversely, the mitochondrial Na^+^/Ca^2+^ exchanger (NCLX) functions to extrude excess Ca^2+^ from the mitochondria to prevent calcium overload. Impaired NCLX function has been shown to exacerbate mitochondrial calcium accumulation, further contributing to cellular injury and necrosis in AP (31). This balance between MCU-mediated calcium influx and NCLX-mediated efflux is critical in maintaining mitochondrial integrity and preventing calcium-induced mitochondrial dysfunction (41). Targeting MCU and NCLX to regulate mitochondrial calcium homeostasis presents a promising therapeutic avenue for mitigating mitochondrial dysfunction in AP.

Under physiological conditions, Ca^2+^ is predominantly stored in the ER, with cytosolic Ca^2+^ levels approximately 10,000 times lower than those in the extracellular fluid (42). Intracellular calcium levels are generally stable under normal conditions, but studies have shown an increase in intracellular Ca^2+^ during AP, which correlates with disease severity (43). In normal physiology, Ca^2+^ acts as a second messenger when released from the ER, triggering the exocrine secretion of zymogen granules (ZG) and promoting ATP production in mitochondria (23, 42). However, the rise in cytoplasmic calcium concentration is transient, as elevated Ca^2+^ is swiftly removed by two ATP-dependent calcium pumps: the sarcoplasmic/endoplasmic reticulum calcium ATPase (SERCA) pumps Ca^2+^ back to the ER, while the plasma membrane Ca^2+^ ATPase (PMCA) expels Ca^2+^ out of the cell, ensuring intracellular calcium balance (44).

**Figure 2:**
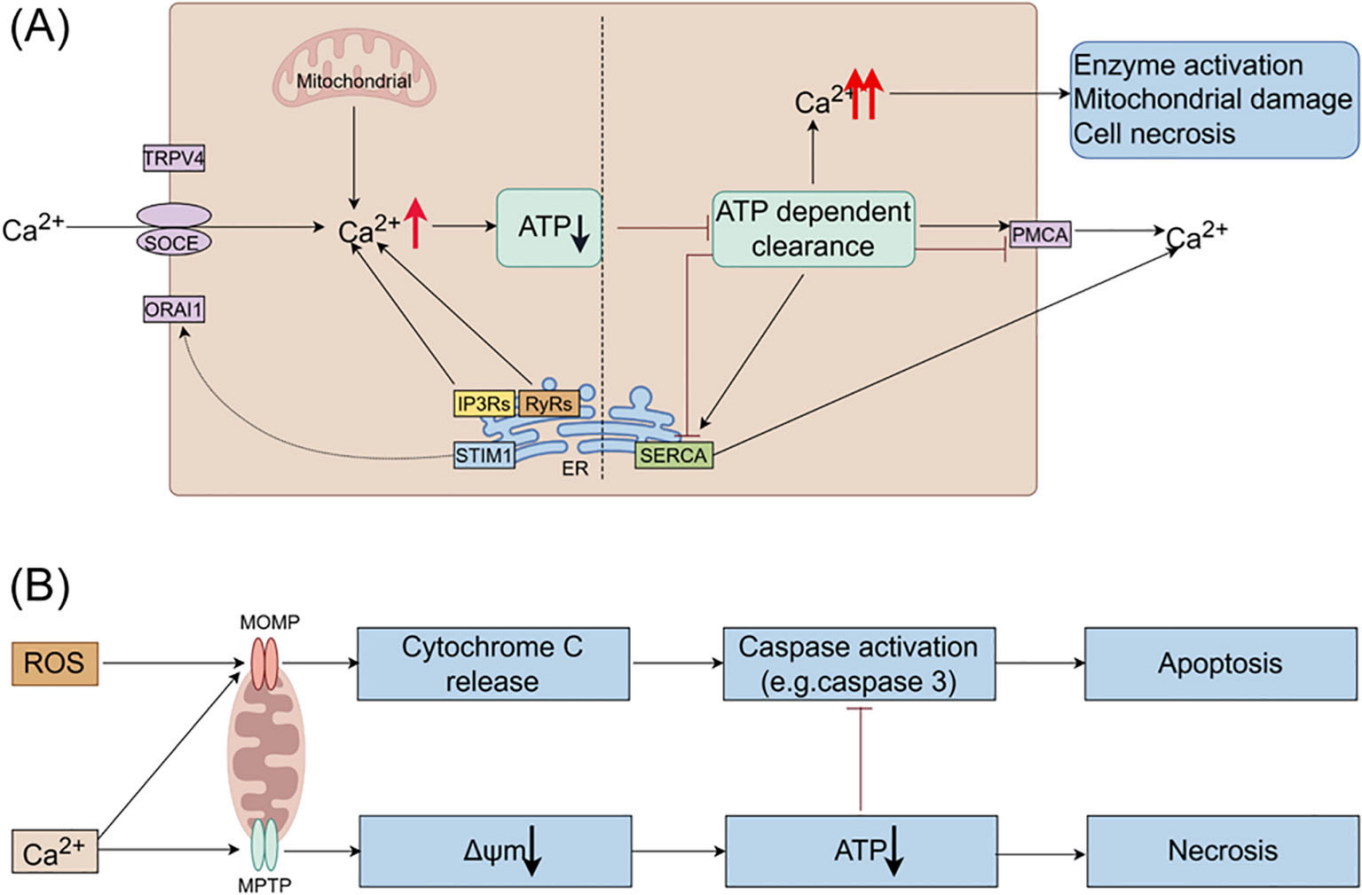
The roles of Ca^2+^ and ROS in mitochondrial pathways of apoptosis and necrosis in pancreatitis. **(A)** In pancreatic acinar cells, intracellular Ca^2+^ overload resulting from pathological Ca^2+^ signaling or inadequate clearance by ATP-dependent mechanisms is a major factor contributing to the development of acute pancreatitis. The regulation of Ca^2+^ signals in pancreatic acinar cells involves various Ca^2+^ channels, such as Ca^2+^ release channels like IP3Rs and RyRs on intracellular stores, SOCE on the cell membrane, and SERCA for refilling intracellular stores. **(B)** Ca^2+^ stimulate the opening of mitochondrial MPTP, resulting in a decrease in mitochondrial membrane potential, ATP depletion, and cell necrosis. ROS promote the release of cytochrome c through MOMP, leading to caspase activation and apoptosis. Additionally, Ca^2+^ itself can trigger the release of cytochrome c and induce cell apoptosis. Moreover, decreased ATP production inhibits caspase activation. Therefore, mitochondrial depolarization not only mediates necrosis but also restricts apoptosis in pancreatitis, elucidating the inverse relationship between acinar cell necrosis and apoptosis observed in experimental pancreatitis models. ATP, adenosine triphosphate; MOMP, mitochondrial outer membrane permeabilization; MPTP, mitochondrial permeability transition pore; IP3Rs, inositol 1,4,5-trisphosphate receptors; ORAI1, Orai calcium release-activated calcium modulator 1; PMCA, plasma membrane Ca^2+^ ATPase; ROS, reactive oxygen species; RyRs, ryanodine receptors; SERCA, sarcoplasmic/endoplasmic reticulum calcium ATPase; SOCE, store-operated Ca^2+^ entry channels; STIM1, stromal interaction molecule 1; TRPV4, transient receptor potential vanilloid 4.

Under pathological conditions, factors such as alcohol, bile acids, and cholecystokinin stimulate the release of Ca^2+^ from the ER, resulting in intracellular calcium overload. This overload is primarily mediated by the inositol 1,4,5-trisphosphate receptors (IP3Rs) and ryanodine receptors (RyRs) located on the ER membrane (see **Figure 2**) (45), evidenced that bombesin can enhance the gene expression of IP3Rs and RyRs, causing calcium overload, while docosahexaenoic acid can inhibit this process, thereby restoring normal calcium signaling within acinar cells (46). Sustained release of Ca^2+^ from the ER depletes calcium stores, triggering stromal interaction molecule 1 (STIM1) to detect the decrease in ER luminal Ca^2+^ levels and activate store-operated calcium entry (SOCE) channels to replenish calcium stores (47). Calcium release-activated calcium channel protein 1 (ORAI1) plays a crucial role in the SOCE channel. STIM1 is responsible for recruiting and activating ORAI1 to initiate the opening of the SOCE channel (47). The activation of this channel exacerbates calcium overload (48). The ORAI1 inhibitor (CM4620) can prevent acinar cell necrosis and reduce local and systemic inflammatory responses in both human pancreatic acinar cells and animal models of AP (49).

Excessive intracellular Ca^2+^ levels trigger the opening of the mitochondrial permeability transition pore (MPTP), resulting in the loss of mitochondrial membrane potential and hindering ATP production (50). Consequently, perigranular mitochondria are unable to regulate the rise in apical Ca^2+^ concentration, leading to the propagation of local Ca^2+^ signaling across acinar cells (51). Reduced ATP production impairs the ability of SERCA and PMCA to eliminate cytoplasmic Ca^2+^, thereby perpetuating the escalation of intracellular Ca^2+^ levels. Prolonged Ca^2+^ overload induces alterations in mitochondrial membrane permeability, mitochondrial impairment, and ultimately, acinar cell necrosis through a destructive cycle.

In addition, ERCP, cholelithiasis, and other factors can also contribute to AP by triggering the Piezo1 channel in response to high pressure in the pancreatic duct, leading to an influx of Ca^2+^ (52). Piezo1, present in various tissues, is selective for Ca^2+^ and can be activated by mechanical pressure, playing a role in pressure-induced AP (53). Research indicates that the activation of Piezo1 channels results in a transient increase in Ca^2+^ levels in acinar cells, followed by the activation of the transient receptor potential vanilloid 4 (TRPV4), leading to sustained Ca^2+^ influx. Knocking out the TRPV4 gene in mice has been shown to prevent both Piezo1 agonist-induced and pressure-induced AP (54).

Given the pivotal role of calcium overload in the pathogenesis of AP, targeting calcium channels represents a promising strategy for early intervention. Understanding the intricate calcium signaling in pancreatic acinar cells can lead to the development of therapeutic approaches aimed at modulating calcium homeostasis to prevent or mitigate AP.

### 3.2 Mitochondrial ATP depletion

Mitochondria are essential for supplying energy for cellular functions through the synthesis of ATP. Dysfunction in mitochondria can result in ATP depletion, leading to various physiological dysfunctions that rely on ATP, such as ZG secretion, Ca^2+^ clearance, and autophagy. Continuous calcium influx can cause intracellular calcium overload, damaging the mitochondrial membrane, opening the MPTP, altering the mitochondrial membrane potential, and ultimately reducing ATP production (50). This decrease in ATP levels inhibits ATP-dependent transport mechanisms like SERCA and PMCA, preventing the removal of intracellular Ca^2+^ and resulting in sustained intracellular calcium overload (see **Figure 2**). This overload can activate digestive ZG, resulting in the pancreatic autodigestion.

Research has demonstrated that elevated levels of non-conjugated chenodeoxycholate (CDC) can lead to mitochondrial damage and ATP depletion, which in turn can directly hinder the secretion of pancreatic duct fluid and bicarbonate (HCO^3−^) (39). Studies have shown that supplementing ATP *in vitro* can prevent damage and dysfunction in acinar cells during AP (55, 56). Furthermore, the MPTP inhibitor cyclosporin A (CsA) and its derivative TRO40303 have been found to decrease acinar cell necrosis and mitochondrial damage, ultimately improving alcoholic AP by inhibiting the opening of MPTP (57). Moreover, early-stage supplementation of high-calorie nutritional support to boost ATP levels may provide a promising approach to mitigating disease progression (58).

### 3.3 Mitochondrial permeability transition pore dysfunction

The mitochondrial permeability transition (mPT) is a process that increases the permeability of the IMM in a Ca^2+^-dependent and cyclophilin D (CypD)-promoted manner, allowing molecules of approximately 1.5 kDa to pass through (59, 60). This phenomenon is regulated by the MPTP, a non-selective channel on the IMM that is sensitive to CsA. Activation of MPTP can occur in response to Ca^2+^ overload, ROS, and other stress signals, leading to cell death through necrosis or apoptosis.

The pore-forming structure of mPT is currently not well understood. Research has indicated that the channel of mPT may involve the voltage-dependent anion channel (VDAC) (61), pro-apoptotic Bcl-2 family members (Bax and Bak) (62–64), phosphate carrier (PiC) (65), and translocator protein (TSPO) (66). While the exact molecular mechanism of MPTP formation remains uncertain, recent studies suggest that mitochondrial F_1_F_0_-ATP synthase, ANT, and the matrix CypD play a role in promoting its transition to a pore-forming conformation (67–69).

MPTP exhibits two distinct opening states: transient and sustained. The transient opening of MPTP regulates various processes including mitochondrial Ca^2+^ efflux (70, 71), ROS signaling (72), cell metabolism (73), and the differentiation of neurons, cardiac muscle, and stem cells (74–76). This physiological process involves the rapid exchange of solutes between the cytoplasm and the mitochondrial matrix, which is crucial for cellular signaling. However, the balance of MPTP opening is delicate and highly regulated. While transient opening of the MPTP supports essential cellular functions, the sustained opening of the MPTP results in mitochondrial swelling and mitochondrial OMM rupture, leading to subsequent apoptosis and necrotic cell death (77). This process is associated with a series of pathologies. Firstly, the increased permeability of the mitochondrial membrane caused by the continuous opening of MPTP results in the disruption of membrane potential and interruption of electron transport, resulting in diminished ATP production. Insufficient ATP affects the energy supply of cells and exacerbates pancreatic cell dysfunction. Secondly, MPTP opening may increase ROS production. When MPTP opens, oxidative substances and free radicals in mitochondria can escape into the cytoplasm, triggering oxidative stress reactions and causing increased cell damage and inflammatory responses. Additionally, increased mitochondrial membrane permeability can activate apoptotic pathways. The release of apoptosis-related proteins such as Cytc from mitochondria into the cytoplasm upon MPTP opening activates the caspase family, ultimately triggering apoptosis (78, 79). Studies have indicated that in pancreatitis, MPTP opening and increased mitochondrial membrane permeability are crucial mechanisms leading to ATP depletion and cellular dysfunction (50). Finally, MPTP opening is linked to the mtDNA release during innate immunity (80). Research has demonstrated that oxidative stress can induce the release of mtDNA through MPTP in rat liver cells (81).

Increased mitochondrial membrane permeability is a significant contributor to the pathogenesis of pancreatitis. This phenomenon results in decreased energy production, elevated ROS levels, and initiation of apoptosis, ultimately exacerbating pancreatic cell injury and triggering inflammatory processes. Consequently, modulating alterations in mitochondrial membrane permeability could offer a promising therapeutic approach for managing pancreatitis.

### 3.4 Oxidative stress

Oxygen free radicals are significant contributors to the pathogenesis of various inflammatory diseases and are crucial in the oxidative stress (OS) mechanism of AP (82). OS occurs when the production of ROS outweighs the capacity of antioxidant defenses, leading to cell damage either directly or by modulating signaling pathways (83). Research has demonstrated that oxygen free radicals are essential in driving the development of AP in an *in vivo* model of experimental AP (56).

The generation of mitoROS primarily occurs during the electron transfer process of the mitochondrial respiratory chain (84). MitoROS production involves several key steps: (1) Complexes I, II, and III of the respiratory chain: Complex I (NADH dehydrogenase) and complex II (succinate dehydrogenase) on the IMM convert H^+^ to coenzyme Q, releasing electrons in the process. These electrons are then transferred to Cytc through complex III. (2) Cytc redox: Cytc passes on the electrons to complex IV of the respiratory chain. (3) Complex IV: Complex IV accepts the electrons and combines them with oxygen to produce water. However, some electrons may escape the respiratory chain during this process and react with molecular oxygen to generate O^2-^ (85, 86). While O^2-^ itself is relatively inactive, it can lead to the formation of more harmful ROS, such as hydrogen peroxide (H_2_O_2_) and hydroxyl radicals (-OH), through subsequent reactions.

In AP, the pancreas releases inflammatory mediators that place mitochondria under sustained high load, leading to damage to the mitochondrial respiratory chain. This dysfunction, along with the excessive accumulation of ROS, is key in the progression of the disease. The impaired mitochondria hinder the activities of MAPK and AKT, resulting in insufficient ATP production to sustain cellular functions and exacerbating ROS accumulation.

ROS is essential in regulating the inflammatory response in AP. This inflammatory response is a key pathological process in pancreatitis, and the overproduction of mitoROS can activate various inflammatory signaling pathways, including NF-κB and NLRP3 inflammasome. Activation of these pathways leads to increased inflammatory cytokines release, including TNFα and IL-1β, thereby perpetuating and exacerbating the inflammatory response (87). Furthermore, mitoROS are implicated in pancreatic cell damage and necrosis in AP. Mitochondrial dysfunction induced by pancreatitis leads to heightened production of mitoROS, causing elevated intracellular oxidative stress. The ROS generated during OS can directly harm cell membranes, nucleic acids, and proteins, ultimately triggering cell apoptosis and necrosis (88–90).

Studies have shown that in AP, mitoROS production initiates cell apoptosis and disrupts insulin secretion function by activating the apoptosis signal-regulated kinase 1 (ASK1) pathway (91). Additionally, mitoROS contribute to the development of pancreatic fibrosis in pancreatitis. The chronic progression of pancreatitis can result in pancreatic fibrosis, with excessive mitoROS production closely linked to fibrosis formation. MitoROS can enhance the activation of pancreatic stellate cells, prompting the synthesis of extracellular matrix and collagen deposition, thus altering pancreatic tissue structure and fostering fibrosis. Moreover, ROS may also affect the stability of mtDNA and the transmission of genetic information, influencing gene expression and function in pancreatic cells. MtDNA is vulnerable to direct attack by ROS, leading to oxidative damage and mutations that disrupt mitochondrial function. This further exacerbates the abnormal state of mitochondria and the generation of ROS, creating a vicious cycle. Studies have shown that in an AP model, mitochondrial function is impaired due to inflammatory reactions, resulting in a significant increase in intracellular ROS levels. These ROS can activate the NF-κB signaling pathway, promoting the production of inflammatory cytokines, leading to the persistence and exacerbation of inflammatory reactions (92). Additionally, excessive ROS production triggers mitochondrial membrane depolarization and the activation of apoptotic signals, causing necrosis and apoptosis of pancreatic cells (93).

The roles and impacts of mitoROS in pancreatitis are diverse. It plays a part in regulating inflammatory responses, causing pancreatic cell damage and necrosis, contributing to pancreatic fibrosis, and disrupting normal pancreatic function through effects on mitochondrial function and cellular gene expression. A comprehensive understanding of how mitoROS functions is crucial for uncovering the pathogenesis of pancreatitis and developing effective treatment approaches.

### 3.5 Impaired autophagy

Autophagy is a fundamental biological mechanism where lysosomes are used to break down macromolecules and damaged organelles. It is controlled by autophagy-related genes (Atg) (94). Through autophagy, cells can eliminate, recycle, and degrade various defective cytoplasmic contents, such as damaged organelles, denatured proteins, or lipids, to prevent ER stress and maintain protein synthesis. This process plays a crucial role in sustaining cell homeostasis (95, 96). Research has indicated that impaired autophagy is also a contributor for the pathogenesis of AP (97).

The process of autophagy involves several steps based on the pathways of substrates entering lysosomes, including macroautophagy, microautophagy, and chaperone-mediated autophagy (CMA). Initially, upon cell stimulation by signals, the UNC-51-like kinase 1 (ULK1) complex is detached from and activated by the mammalian target of rapamycin complex 1 (mTORC1) through a cascade reaction, thereby initiating autophagy (98). Subsequently, the activated ULK1 complex recruits the Beclin1-Vps34 complex, resulting in the creation of double-layered intracellular membrane vesicles. These vesicles, containing various Atg-encoded proteins and ubiquitination receptors (such as p62), elongate with the help of microtubule-associated-protein light-chain-3-II (LC3-II), encapsulate waste products, and give rise to autophagosomes (99, 100). Finally, the autophagosomes merge with lysosomes, a process facilitated by lysosome-associated membrane proteins (LAMPs). Upon fusion, lysosomal cathepsins like CTSB and CTSL degrade and clear the waste material, allowing for recycling (101, 102).

Autophagy impairment in AP acinar cells is characterized by elevated autophagosome production and decreased lysosomal degradation, leading to heightened inflammatory cell infiltration, acinar cell necrosis, and apoptosis (103). Studies have shown that impaired autophagy contributes to acinar cell vacuolization and zymogen activation, triggering AP development (104). Lysosome-associated membrane protein-2 (LAMP-2), a lysosomal membrane protein abundant in pancreatic tissue, plays a crucial role in autophagosome-lysosome fusion (105). In an acute necrotizing pancreatitis rat model, decreased levels of LAMP-2 led to the accumulation of undegraded material in abnormally enlarged vacuoles within acinar cells, indicating impaired autophagy (106). Knockout of LAMP-2 hindered autophagosome-lysosome complex formation, resulting in the buildup of autophagosomes, limited zymogen granule degradation, and abnormal trypsinogen activation, exacerbating AP (107, 108). Additionally, knockout of Atg5 and Atg7 in mouse models of AP impaired autophagy and worsened the disease (109). LC3, the earliest autophagy marker identified, is closely linked to autophagosome abundance, serving as a key indicator of autophagy activity (110). Moreover, p62, a well-studied autophagy substrate, plays a critical role in mitochondrial clearance (111). The impairment of autophagy efficiency in AP can be observed through elevated levels of pancreatic autophagy markers LC3-II and autophagy substrate p62/SQSTM1, as well as an increase in ubiquitinated protein accumulation (112). Research indicates that in AP, interventions targeting the modulation of LC3 and p62/SQSTM1 expression can enhance autophagy and mitigate pathological harm to the pancreas (113, 114).

The process that selectively eliminates damaged mitochondria is known as mitophagy. Mitophagy plays a crucial role in the pathogenesis of pancreatitis by regulating cellular processes that include inflammation and cell death. Impairments in mitophagy may result in insufficient removal of damaged mitochondria, leading to exacerbated pathological responses in pancreatitis, such as a shift in the balance between apoptosis and necrosis, which are pivotal in determining the severity of pancreatitis (115). When mitochondria lose their membrane potential, it can trigger the onset of autophagy, particularly mitophagy, creating a harmful cycle (116).

Mitophagy is primarily regulated by several key pathways, including the PINK1/Parkin pathway, the Bnip3/Nix-mediated pathway, and the FUNDC1-mediated pathway. In the PINK1/Parkin pathway, PINK1 accumulates on the outer mitochondrial membrane when the membrane potential is lost, leading to the recruitment of the E3 ubiquitin ligase Parkin. Parkin ubiquitinates mitochondrial surface proteins, marking them for degradation via the autophagosome (117). This pathway plays a critical role in mitochondrial quality control, ensuring the removal of damaged mitochondria to maintain cellular homeostasis, especially in high-energy-demanding cells, such as neurons and acinar cells (118). Studies also highlight the role of the Bnip3 and Nix proteins in facilitating mitophagy by promoting the interaction between damaged mitochondria and the autophagy machinery (119). Furthermore, Bnip3 can suppress PINK1 cleavage, enhancing the accumulation of full-length PINK1, which is necessary for efficient Parkin recruitment and mitophagy activation (120). The FUNDC1-mediated mitophagy pathway, particularly active under hypoxic conditions, plays a critical role in maintaining mitochondrial homeostasis. FUNDC1 is a mitochondrial outer membrane protein that interacts with LC3 to facilitate the clearance of damaged mitochondria (121). Hypoxia and mitochondrial dysfunction trigger the dephosphorylation of FUNDC1, enhancing its interaction with LC3 and promoting mitophagy, which helps protect cells during stress conditions such as AP (122). Additionally, mitochondrial damage can trigger inflammatory responses, worsening the situation.

Therefore, suppressing the formation of autophagosomes can help mitigate damage caused by autophagy, decrease the activation of digestive enzymes, and provide some relief from AP (123). Pharmacological manipulation of autophagy presents a viable avenue for treating AP. Research indicates that IL-22 can mitigate autophagosome formation via the Beclin1 pathway, thus alleviating the severity of AP (124). The disaccharide trehalose has demonstrated enhanced autophagic efficiency and reduced pancreatic damage in animal models of AP, suggesting its potential as a therapeutic agent (2). Moreover, lycopene has shown promise in ameliorating AP severity in mice by modulating autophagy; however, further investigation is warranted to elucidate its mechanism of action and therapeutic efficacy in human pancreatitis (125). Notably, studies suggest that statins may lower the incidence of AP and enhance prognosis (126, 127). In a rat model of AP, simvastatin restored autophagic flux by promoting autophagosome-lysosome fusion, thereby mitigating mitochondrial damage and inflammatory responses (101). This further supports the notion that promoting complete mitophagic flux, rather than just the initiation of mitophagy, could be critical in mitigating pancreatic inflammation and damage in AP. Thus, understanding the specific mechanisms of mitophagy, particularly the regulation of PINK1/Parkin, Bnip3/Nix and FUNDC1 pathways, may lead to novel therapeutic targets in AP (128). Additionally, the balance mitophagy maintains between apoptosis and necrosis underscores its significance in the pathology of pancreatitis, particularly in relation to inefficient lysosomal function and autophagy impairment (115). Consequently, exploring the function and control of autophagy in AP, as well as developing interventions to restore autophagy in AP acinar cells, are poised to be pivotal areas of future research.

### 3.6 Mitochondrial regulation of cell death (apoptosis, necrosis and pyroptosis)

Besides supplying energy to cells, mitochondria regulate necrosis and apoptosis of acinar cells through changes in mitochondrial membrane permeability (MMP) (19). Excessive Ca^2+^ accumulation in acinar cells triggers the opening of the MPTP in the IMM, leading to loss of mitochondrial membrane potential, impaired ATP production, and eventual necrosis (see **Figure 2**). The Bcl-2 family proteins play a key role in mediating the release of Cytc and regulating apoptosis by controlling mitochondrial outer membrane permeability (MOMP) (129). During AP, both necrosis and apoptosis occur simultaneously in acinar cells.

Acinar cell death is a key pathological response in AP. Research has demonstrated that in animal models of AP, the severity is directly proportional to the extent of necrosis and inversely proportional to the level of apoptosis. Furthermore, inducing acinar cell apoptosis has been shown to decrease the severity of necrosis and AP, whereas inhibiting apoptosis with caspase inhibitors like XIAP can worsen necrosis (130). Hyperbaric oxygen therapy has been found to alleviate disease severity by promoting acinar cell apoptosis and reducing necrosis (131). Using isolated mitochondria and acinar cells, it was found that increased expression of the Bcl-2 protein can decrease pancreatic acinar cell necrosis by preventing mitochondrial depolarization and subsequent ATP depletion (132). Additionally, silencing the hypoxia-inducible factor 1α (HIF1α) gene can enhance intracellular energy balance by preserving mitochondrial homeostasis, reducing necrosis, and promoting apoptosis, ultimately mitigating the inflammatory response in AP (133).

Furthermore, mitochondrial dysfunction is recognized as a significant factor in the pyroptosis of pancreatic acinar cells during AP. Mitochondrial damage can result in elevated intracellular Ca^2+^ levels, which in turn induces OS within the cell. This OS may lead to cell membrane rupture and subsequent pyroptosis (134, 135). Lieberman et al. demonstrated that the N-terminal pore-forming fragment of Gasdermin D (GSDMD-NT) targets mitochondria; during the pyroptosis process, GSDMD-NT rapidly damages both IMM and OMM leading to reduced mitochondrial numbers, mitophagy, ROS, loss of transmembrane potential, attenuated oxidative phosphorylation and release of mitochondrial proteins and DNA from the matrix and intermembrane space (136). Research has indicated a reciprocal relationship between mitochondrial dysfunction and inflammasome activation. ROS released from mitochondria can activate the NLRP3 inflammasome, thereby facilitating the onset of pyroptosis. This process establishes a vicious cycle: mitochondrial damage leads to inflammasome activation, which in turn exacerbates mitochondrial damage (137, 138).

Recent research has identified that certain cytokines, such as IL-37, may exert a protective effect in cases of AP. IL-37 has been shown to inhibit the pyroptosis of damaged acinar cells, a mechanism that appears to be linked to its ability to inhibit the activation of the NLRP3 inflammasome. By specifically removing GSDMD from the pancreas, researchers demonstrated that the protective effect of IL-37 was neutralized, indicating that GSDMD plays a crucial role in the pyroptosis process (139). Additionally, drugs targeting pancreatic acinar cell pyroptosis, such as high-density lipoprotein (HDL) and apoA-I, have been found to inhibit both the activation of the NLRP3 inflammasome and pyroptosis in acinar cells (135). This suggests a novel therapeutic approach for the treatment of AP, potentially alleviating the condition by modulating the inflammatory response. Besides, cold-inducible RNA binding protein (CIRP) has been implicated in inducing mitochondrial dysfunction and pyroptosis in pancreatic acinar cells, indicating that blocking CIRP may represent an effective strategy for treating AP (140).

### 3.7 Mitochondrial dynamics imbalance

**Figure 3:**
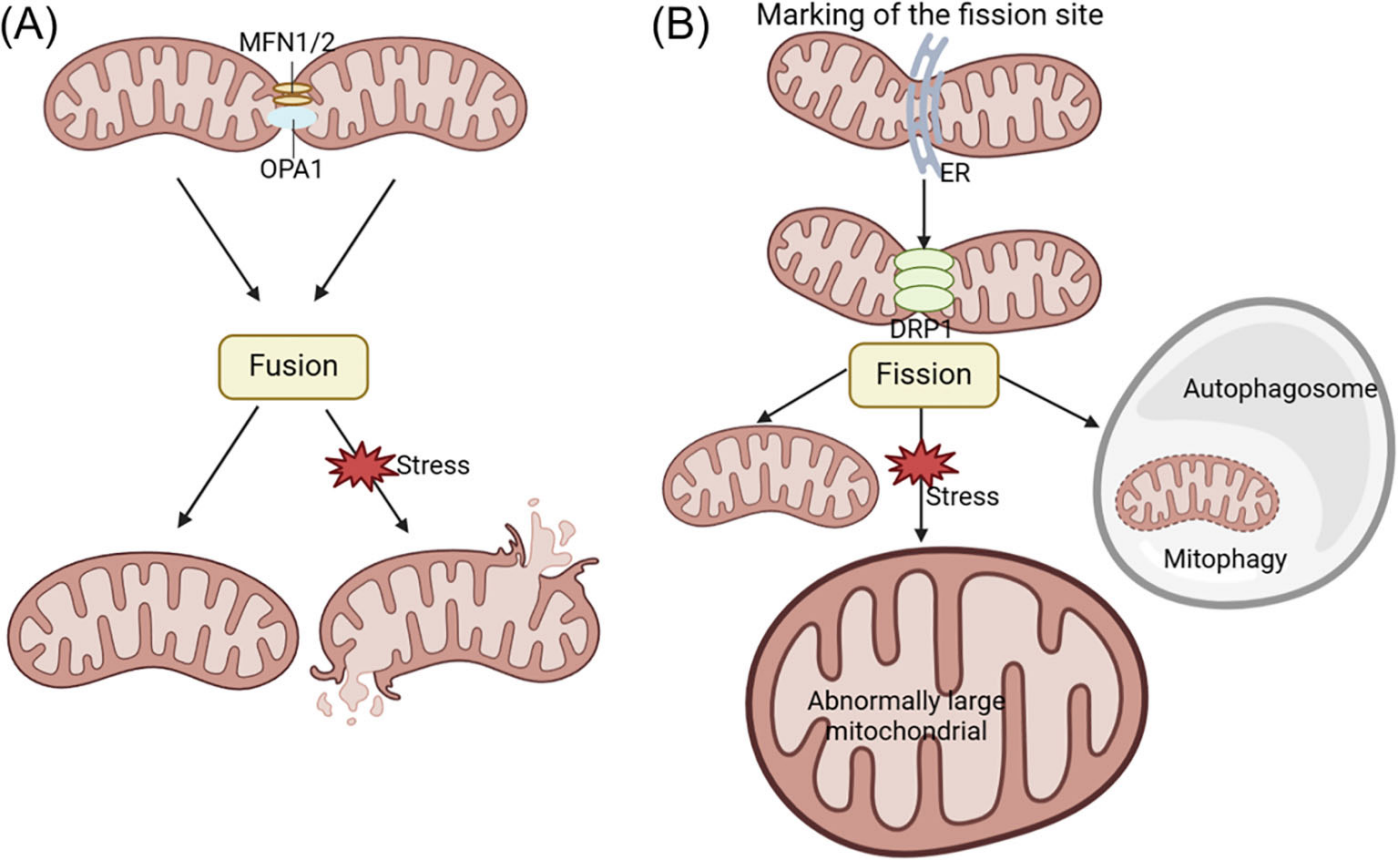
The changes of acinar cell mitochondrial dynamics in pancreatitis. Mitochondrial fusion and fission processes play a crucial role in promoting ATP production and maintaining quality control within the cell. Fusion involves the mixing of mitochondrial contents, while fission generates new healthy mitochondria and facilitates the removal of defective mitochondria through mitophagy. Mitochondrial fusion is primarily regulated by MFN1/2 and OPA1, while mitochondrial fission is mainly controlled by DRP1 regulation. Abnormal mitochondrial fusion can cause fragmentation **(A)**, while disorders in mitochondrial fission can lead to the formation of oversized mitochondria **(B)**. DRP1, dynamin-related protein 1; ER, endoplasmic reticulum; MFN1/2, mitofusin-1/2; OPA1, optic atrophy 1.

Mitochondrial dynamics involves the continuous fusion and fission processes that mitochondria undergo to regulate the shape, number, and distribution. This process is crucial for maintaining mtDNA, ATP production, calcium homeostasis, signal transduction, and apoptosis (141, 142). Imbalances in mitochondrial fission and fusion often result in structural changes and dysfunction within mitochondria. Abnormal mitochondrial fusion can cause fragmentation, while impairments in mitochondrial fission can result in the oversized mitochondria formation. These imbalances in mitochondrial dynamics can disrupt the intracellular environment, cause cellular damage, and even result in cell death. Mitochondrial fusion is primarily regulated by mitofusin-1/2 (MFN1/2) and optic atrophy 1 (OPA1), while mitochondrial fission is mainly controlled by dynamin-related protein 1 (DRP1) (see **Figure 3**) (143–146).

Mitochondrial dysfunction during AP development is characterized by disruptions in mitochondrial dynamics, as evidenced by variations in OPA1 and DRP1 expression and distinct ultrastructural features such as mitochondrial fission, elongation, and mitophagy (147). Research has shown that the TAK-242, a novel toll-like receptor 4 (TLR4) antagonist, can protect taurocholate-induced AP acinar cells in mice. TAK-242 was found to prevent changes in protein expression associated with mitochondrial dynamics. Specifically, the levels of OPA1 and MFN1 were elevated compared to the control group of normal mice, while DRP1 expression decreased. These results demonstrate that TAK-242 can enhance cellular function in AP by modulating mitochondrial dynamics and reducing taurocholate-induced cytotoxicity (148).

### 3.8 Mitochondrial DNA integrity and dysfunction

The role of mtDNA in pancreatitis spans several critical aspects, ranging from its contribution to the pathogenesis of the disease to its potential as a biomarker for diagnosing disease severity. Numerous studies have highlighted how mitochondrial dysfunction, often influenced by mtDNA alterations, can significantly impact the progression of pancreatitis.

Mutations and dysfunctions in mtDNA have been associated with various pancreatic diseases. For instance, a patient with Kearns-Sayre syndrome, a disorder linked to mtDNA-related mitochondrial dysfunction, experienced recurrent episodes of AP, underscoring the direct impact of mtDNA on pancreatitis (32). Furthermore, mitochondrial dysfunction, including impairments in mitochondrial network dynamics, cristae morphology, and mtDNA nucleoid structure, plays a crucial role in diseases like type 2 diabetes, which affect pancreatic β-cells by disrupting glucose sensing and regulation (149). Although these mechanisms are not directly linked to pancreatitis, they emphasize the broader role of mtDNA in maintaining pancreatic health.

Mitochondrial complex I deficiency has been shown to promote pancreatic α-cell proliferation in models of premature aging, suggesting that mtDNA mutations may have compensatory or pathogenic roles in pancreatic tissue (150). Additionally, VMP1-dependent selective mitophagy and mitochondrial fragmentation, driven by mtDNA, act as protective cellular mechanisms in pancreatitis, highlighting mtDNA’s involvement in cellular responses to the disease (147). Moreover, circulating mtDNA has emerged as a potential biomarker for predicting the severity of AP, indicating that its role extends beyond cellular functions to include disease diagnosis and prognosis (151). This is supported by evidence that mtDNA contributes to mitochondrial dysfunction and induces apoptosis in acinar cells, playing a key role in the pathogenesis of pancreatitis (152).

Beyond its pathogenic influence, mtDNA also plays a role in shaping the course of pancreatitis through mechanisms that regulate cell survival, such as balancing apoptosis and necrosis, which are pivotal in determining the severity of the disease (115). The balance between these processes highlights the critical importance of mitochondrial health in influencing the progression of pancreatic diseases. While numerous studies have identified genetic mutations in nuclear genes, including PRSS1, PRSS2, SPINK1, CFTR, CTRC, CASR, and CLDN2, which are strongly associated with different forms of pancreatitis (153, 154), studies have also explored the potential role of mitochondrial DNA (mtDNA) mutations in the disease’s pathogenesis. For example, the A3243G mutation in mitochondrial DNA, especially within the tRNALeu(UUR) gene, has been identified as a contributing factor to the increased prevalence of diabetes and notably recurrent pancreatitis within a selected familial grouping (155). This suggests that mtDNA mutations may predispose individuals to pancreatitis by impairing pancreatic β-cell function and exocrine regulation. Additionally, a case study reports the first case of chronic pancreatitis associated with mitochondrial encephalopathy, linked to the A8344G mtDNA mutation, highlighting the potential role of mitochondrial dysfunction in recurrent pancreatitis (156). Furthermore, the mtDNA nt7778 G-to-T polymorphism does not exacerbate cerulein-induced AP in mice but may accelerate the progression of autoimmune-like lesions after tissue damage, particularly in older mice, indicating its potential role for the polymorphism in autoimmune disease susceptibility following pancreatic injury (157).

## 4 Mitochondrial-related signaling pathways in acute pancreatitis

**Figure 4:**
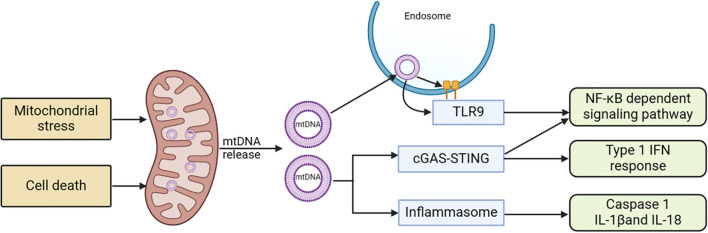
Mitochondria-related signaling pathways and acute pancreatitis. Stress stimulation or cell death leads to the release of mtDNA into the cytoplasm. This mtDNA can initiate various pro-inflammatory signaling pathways either through endosomally localized TLR9, cytoplasmic cGAS-STING, or cytoplasmic inflammasomes. TLR9 binds to mtDNA in endosomes, activating an NF-kB-dependent pro-inflammatory signaling cascade. cGAS detects mtDNA in the cytoplasm and triggers STING, located in the ER, resulting in an interferon response. Inflammasome activity dependent on mtDNA leads to caspase-1 activation or the production of pro-inflammatory IL-1 and IL-18 precursors. cGAS, cyclic GMP-AMP synthase; ER, endoplasmic reticulum; mtDNA, mitochondrial DNA; STING1, stimulator of interferon response cGAMP interactor 1; TLR9, Toll-like receptor 9.

Mitochondrial dysfunction can cause the release of various components and products, which can trigger inflammatory responses when they accumulate in the cytoplasm or extracellular environment, potentially leading to cell death. Multiple signaling pathways that initiate inflammatory responses as a result of mitochondrial dysfunction have been discovered, particularly cyclic GMP-AMP synthase (cGAS) - Stimulator of Interferon Genes 1 (STING1) and inflammasome signaling pathways (see **Figure 4**) (158).

### 4.1 cGAS–STING1 signaling pathway

The cGAS-STING is a molecular signaling pathway that involves two proteins: cGAS activated by mtDNA and STING1 (159). cGAS, a cytoplasmic double-stranded DNA (dsDNA) sensor protein, catalyzes the formation of cGAMP (160). Acting as a second messenger that triggers inflammatory responses, cGAMP activates STING1. When mitochondrial outer membrane permeability (MOMP) or other forms of mitochondrial dysfunction led to the entry of mtDNA into the cytoplasm, cGAS signaling is promoted, a process that is hindered by apoptotic caspases (161, 162). In situations where apoptotic caspase activation is limited, mtDNA tends to interact with cGAS and STING1, subsequently resulting in the initiation of type I interferon (IFN) response. Upon exiting the mitochondria, mtDNA can activate cGAS through pores formed by BCL-2 related proteins like BAX and BAK1, or through the permeability transition pore complex (PTPC). This activation leads to the promotion of the STING1 signaling pathway and the expression of inflammatory mediators such as IFN-β1, IL-6, and TNFα (163, 164). While this system usually prevents unnecessary cGAS activation in normal conditions, it retains the ability to trigger inflammatory responses when needed. Numerous studies have demonstrated that mtDNA can strongly induce inflammation via cGAS and STING1, particularly when apoptotic caspase activation is limited (165).

Upon activation, STING1 undergoes a conformational change and translocates to the Golgi apparatus, where it activates downstream kinases, including TBK1 (TANK-binding kinase 1) and IKK (IκB kinase). This results in the phosphorylation and activation of the transcription factors IRF3 and NF-κB (166, 167). Once activated, NF-κB translocates to the nucleus and promotes the transcription of genes encoding inflammatory mediators, including tumor necrosis factor-α (TNF-α), interleukins (e.g., IL-1β, IL-6), and chemokines (168). These mediators recruit and activate immune cells in the pancreas, exacerbating the inflammatory response. That is, the pathway induces a positive feedback loop by expressing adhesion molecules and receptors that enhance immune cell infiltration into the pancreas (169). This amplifies the local inflammatory response, potentially leading to pancreatic tissue damage and necrosis.

### 4.2 Inflammasome signaling pathway

MtDNA and ROS can induce inflammasome activation. In addition to serving as an effective cGAS stimulant, cytosolic mtDNA can trigger inflammasome activation (170). The inflammasome signaling pathway demonstrates that after being released from dysfunctional mitochondria, mtDNA and ROS activate caspase-1, leading to the secretion of IL-1β and IL-18 (171, 172). The electron transport chain (ETC) affects inflammasome activation independently of ROS, maintaining cellular ATP availability through phosphocreatine (173). Here, mtDNA and mitoROS act as the primary DAMPs for inflammasome activation, interacting at multiple nodes in the molecular mechanisms regulating regulated cell death (RCD), thereby significantly influencing RCD (174).

### 4.3 Other inflammatory pathways

Mitochondrial DNA and other mitochondrial components can also activate inflammatory responses through various PPRs (175). The Toll-like receptor family (TLR) is responsible for detecting a wide range of bacterial signatures to initiate innate immunity. TLR9 is primarily found in monocytes, macrophages, plasmacytoid dendritic cells, and B lymphocytes. While TLR9 is initially located on the ER in its inactive state, it recognizes DNA in endolysosomes (176, 177). TLR9 can interact with mtDNA in endosomes, leading to the initiation of an NF-κB -dependent pro-inflammatory signaling pathway (178).

## 5 Therapeutic strategies for mitochondrial damage in acute pancreatitis

Current research is exploring various strategies to protect mitochondrial function, including the use of mitochondrial pharmacoprotection and mitochondrial transplantation.

### 5.1 Mitochondrial transplantation

**Figure 5:**
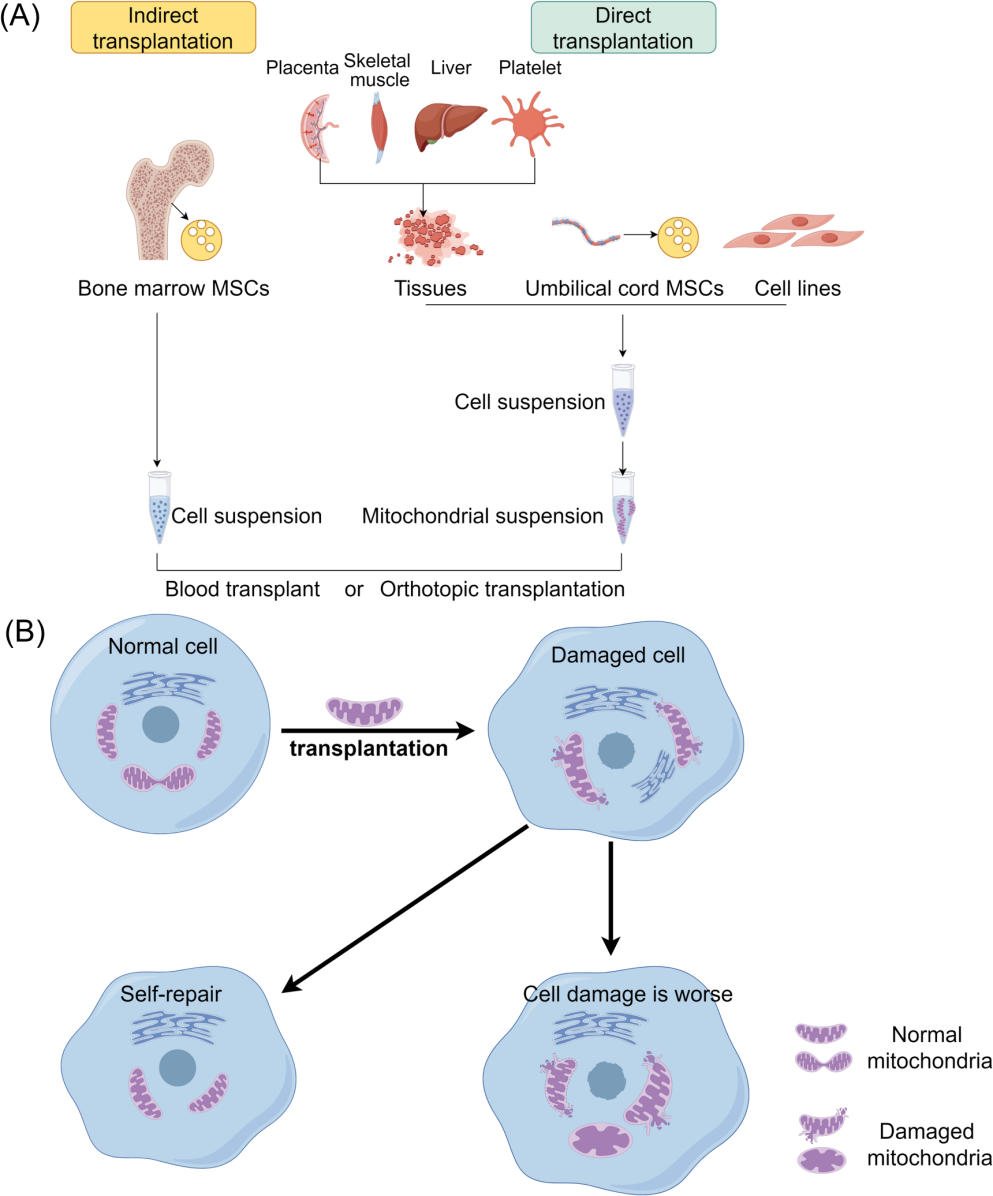
The therapeutic strategies of mitochondrial transplantation. **(A)** Mitochondrial transplantation methods include two categories: direct transplantation and indirect transplantation. Purified mitochondria or vectors carrying mitochondria may be introduced into recipient cells via blood injection or through *in situ* injection. **(B)** Mitochondria derived from healthy cells are transplanted into damaged cells to enhance their function and promote the growth of the recipient cells. MSCs, Mesenchymal stem cells.

Mitochondrial transplantation (MT) involves injecting isolated mitochondria into damaged tissues or organs, or into the bloodstream, to provide therapeutic benefits. In cell culture, it refers to injecting isolated mitochondria for co-incubation with cultured cells to study the effects of mitochondrial transplantation (179, 180). In 2009, Mccully et al. initially documented the significant cardioprotective effect of injecting mitochondria into the ischemic region of the rabbit heart (181). Subsequently, mitochondrial transplantation technology has demonstrated therapeutic benefits in various tissue and organ injuries, including the heart, brain, nervous system, lungs, kidneys, liver, skeletal muscles, and skin, as well as in conditions such as inflammation and tumors (see **Figure 5**) (182–184).

The mechanism of action of mitochondrial transplantation involves both internalization and non-internalization mechanisms (183). Internalization refers to the process of mitochondria entering cells, while the corresponding mechanism is known as internalization mechanism (185). The observation that the number of mitochondria entering cells is small yet the effects are rapid suggests the existence of a non-internalization mechanism, where mitochondria may not need to enter cells to exert their effects (186). Recent research supports that internalized mitochondria can trigger mitophagy through the PINK1-Parkin signaling pathway, a process that clears damaged mitochondria, promotes mitochondrial biogenesis, and restores ATP production. These effects enhance cellular energy metabolism and resilience, particularly in stress conditions (187). Therefore, the mechanism of mitochondrial transplantation action can be categorized into internalization and non-internalization mechanisms. The therapeutic efficacy of the internalization mechanism is rooted in the mitochondria’s ability to produce ATP, while the non-internalization mechanism is based on the idea that mitochondria can interact with specific substances on the cell surface to transmit information and stimulate cellular self-preservation (188, 189).

Mitochondrial transplantation has emerged as a promising therapeutic strategy for AP by addressing the critical role of mitochondrial dysfunction in disease pathogenesis. Mitochondrial dysfunction, a hallmark of AP, contributes to impaired energy metabolism, oxidative stress, and cellular injury, exacerbating disease progression (35). Early studies have identified mitochondrial impairment in pancreatic tissues during AP, underscoring the potential of targeted mitochondrial therapies to mitigate damage and improve outcomes. Recent advancements in mitochondrial transfer mechanisms have opened new avenues for treating AP. Research in vascular biology demonstrates that perivascular mesenchymal stem cells (MSCs) can transfer mitochondria to neighboring endothelial cells (ECs) via tunneling nanotubes (TNTs), enhancing bioenergetics and cellular recovery (187). Interestingly, this therapeutic effect does not rely on the functionality of transferred mitochondria but rather on their ability to trigger mitophagy, facilitating the removal of dysfunctional mitochondria and enhancing mitochondrial turnover.

Although mitochondrial transplantation has not been specifically reported in pancreatitis, it has shown therapeutic effects in improving liver damage and preventing liver fibrosis. In a mouse model of liver injury induced by CCl4, injecting mitochondria from healthy mouse livers intravenously can promote ATP generation, reduce free radical damage, significantly improve liver function, and prevent tissue fibrosis (190). Additionally, artificial mitochondrial transfer (AMT), involving the direct delivery of mitochondria into damaged cells, has shown potential in addressing the energy crisis caused by mitochondrial damage in pancreatic cells (191). Studies reveal that transplanted mitochondria, even when depolarized or mtDNA-free, can effectively trigger mitophagy, leading to enhanced cellular energy metabolism and survival (187, 191). This mechanism holds particular therapeutic relevance in AP, where mitochondrial dysfunction exacerbates acinar cell injury and inflammation.

### 5.2 Mitochondrial pharmacoprotection

**Figure 6:**
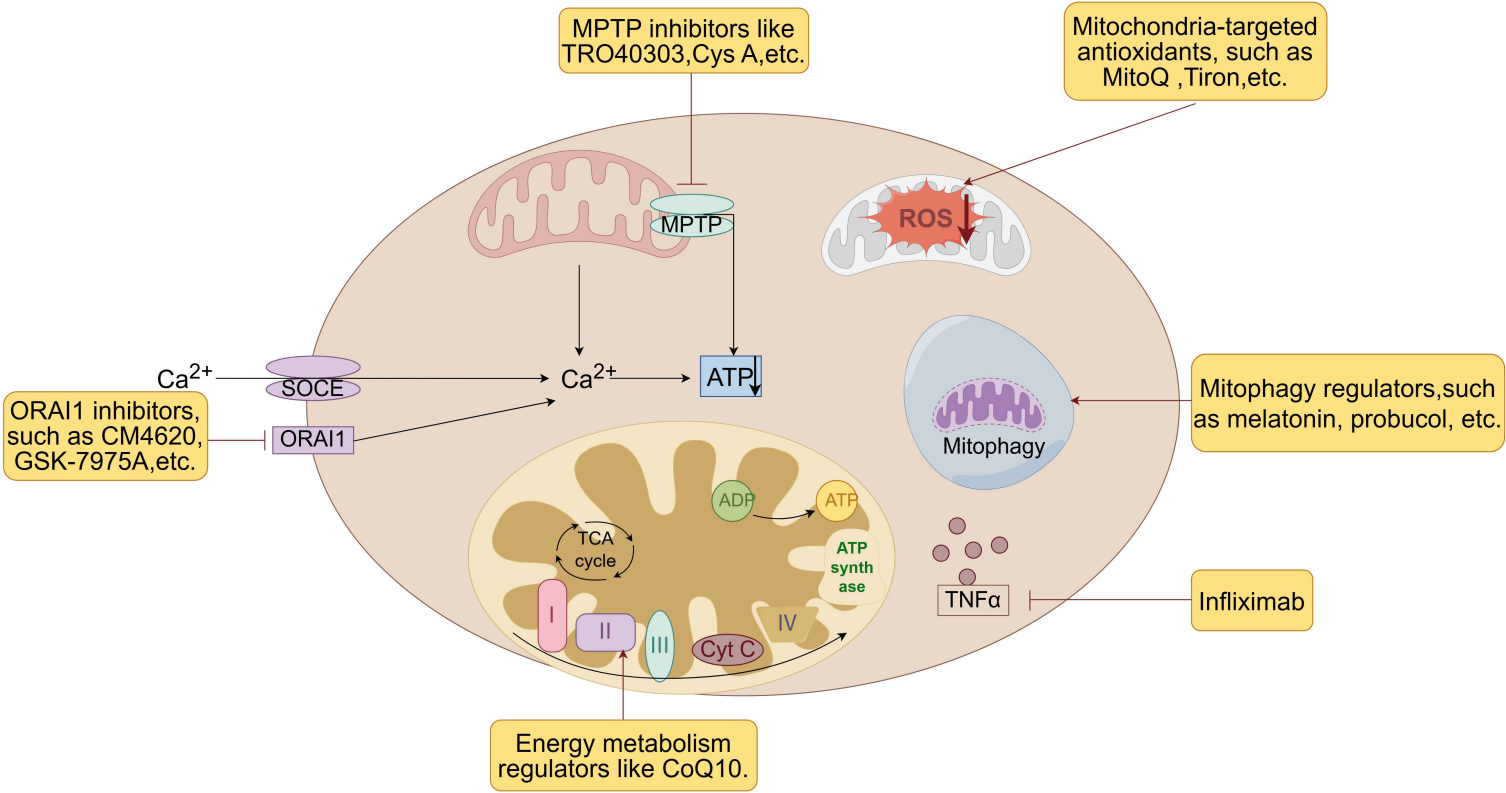
Therapeutic strategies of mitochondrial pharmacoprotection. Mitochondrial pharmacoprotection is a therapeutic strategy aimed at protecting against pancreatitis by modulating mitochondrial function and attenuating mitochondrial damage. These medications include antioxidants, mitochondrial membrane permeability regulators, energy metabolism regulators, mitophagy regulators, anti-inflammatory drugs and drugs for mitochondrial function repair. Cys A, Cyclosporin A; CoQ 10, Coenzyme Q10; MPTP, mitochondrial permeability transition pore; ORAI1, Orai calcium release-activated calcium modulator 1; ROS, reactive oxygen species; SOCE, store-operated Ca2+ entry channels; TNFα, tumor necrosis factor-α.

Mitochondrial pharmacoprotection is a therapeutic strategy aimed at protecting against pancreatitis by modulating mitochondrial function and attenuating mitochondrial damage. Given the significant role of mitochondria in the pathogenesis of pancreatitis, pharmacological interventions targeting these organelles have the potential to reduce inflammatory responses, cellular damage, and disease progression. These medications include antioxidants, mitochondrial membrane permeability regulators, energy metabolism regulators, anti-inflammatory drugs and drugs for mitochondrial function repair (see **Figure 6**).

#### 5.2.1 Antioxidants

Mitochondrial dysfunction is frequently associated with elevated oxidative stress levels, leading to cellular damage and exacerbating the inflammatory response (192). Utilizing antioxidants, particularly those targeted specifically to mitochondria, can offer specialized protection to these organelles and shield them from oxidative stress-induced harm.

Mitochondria-targeted antioxidants, such as MitoQ and Tiron, are a type of drug that specifically target mitochondria to provide antioxidant effects and offer superior protection against mtDNA damage when compared to non-targeted antioxidants like resveratrol, curcumin, and N-acetylcysteine (193, 194). These antioxidants penetrate the mitochondrial phospholipid bilayer and neutralize ROS at the core source, and also have substantial protective effects against damage caused by AP. For instance, Epigallocatechin gallate (EGCG) derived from green tea can effectively decrease L-arginine-induced AP and subsequent lung injury in mice by inhibiting the activation of NLRP3 inflammasome (195). This protective mechanism is thought to involve the elimination of mitoROS and its oxidation product OX-mtDNA (196).

In addition to mitochondria-targeted antioxidants, vitamins also exhibit antioxidant properties and are inversely associated with AP (197). Vitamin B12, acting as an allosteric activator of cysteine-β-synthase (CBS), has been shown to decrease edema, inflammation, and necrosis in experimental pancreatitis. This beneficial effect may be attributed to vitamin B12-mediated enhancement of mitophagy, leading to repair of mitochondrial function (198). Lycopene, a potent antioxidant, has also been demonstrated to offer protection against AP (122). Research indicates that lycopene can mitigate oxidative stress via the JNK pathway and safeguard pancreatic acinar cells from injury (199). Furthermore, lycopene has been shown to have a protective effect in rat models of experimental AP induced by caerulein (200). However, the potential limitations of these agents, such as limited bioavailability and toxicity at higher doses, must be carefully evaluated in future studies.

#### 5.2.2 Mitochondrial membrane permeability regulator

Regulation of MMP plays a crucial role in mitigating abnormal enzyme activation and mtDNA damage. Cyclosporin A, an immunosuppressant, effectively inhibits the opening of MPTP, thereby decreasing mitochondrial membrane permeability. For instance, experimental research has demonstrated the neuroprotective properties of cyclosporine A in alleviating brain damage in rats afflicted with acute necrotizing pancreatitis (201). However, despite its efficacy, cyclosporin A is known for its severe side effects, such as nephrotoxicity and immunosuppression, which limit its clinical application in treating pancreatitis.

Consequently, alternative MPTP inhibitors like TRO40303, which lack these severe side effects, are being investigated as more suitable therapeutic options. TRO40303, a novel MPTP inhibitor, interacts with mitochondrial extraporters, delaying MPTP opening independently of CypD, and reducing membrane potential loss and necrosis in pancreatic acinar cells, ultimately providing systemic protection in AP (57). Studies in murine and human pancreatic acinar cells demonstrated that TRO40303 significantly reduced mitochondrial membrane potential loss, cytosolic calcium overload, and necrotic cell death caused by pancreatic toxins, including taurolithocholate sulfate and cholecystokinin. In animal models of AP, TRO40303 decreased serum amylase, pancreatic trypsin, and inflammation scores, indicating its potential for broad protective effects in AP. Moreover, its ability to reduce mitochondrial dysfunction in experimental models of bile acid and alcohol-induced pancreatitis suggests it could be beneficial in preventing AP exacerbations due to mitochondrial injuries (57). Further studies are required to fully assess its efficacy and safety in clinical settings. However, the promising results obtained so far point toward TRO40303 as a viable candidate for mitochondrial-targeted pharmacotherapy in AP.

Furthermore, CypD inhibitors have shown promise in mitigating necrosis in both mouse and human acinar cells, improving the severity of AP by safeguarding mitochondrial integrity (202).

#### 5.2.3 Calcium modulator

ORAI1, an essential component of the SOCE pathway, plays a crucial role in calcium influx, which is critical for cellular functions and the inflammatory response during pancreatitis. Calcium overload is a key factor in mitochondrial dysfunction and cellular injury in AP (203), making ORAI1 a valuable target for therapeutic intervention. Targeted inhibition of ORAI1 has emerged as a promising therapeutic approach to mitigate the effects of pancreatitis. By preventing excessive calcium influx through the SOCE pathway, ORAI1 inhibitors help maintain intracellular calcium balance, thereby reducing the risk of mitochondrial damage and acinar cell death.

In pancreatitis, the pathological rise in acinar cell cytosolic Ca^2+^ levels mediated by ORAI1 leads to mitochondrial dysfunction and cell death, exacerbating inflammation and tissue injury. The use of ORAI1 inhibitors, such as CM4620, GSK-7975A and CM_128, has been suggested as a therapeutic approach, effectively reducing cytosolic Ca^2+^ overload in pancreatic cells and mitigating the inflammatory response associated with pancreatitis (204, 205). Additionally, the strategy of inhibiting ORAI1 has been found to be significantly more effective when administered early after the onset of pancreatitis, underscoring the importance of timing in therapeutic intervention to maximize efficacy (205).

Furthermore, neutrophil-specific inhibition of the ORAI1 calcium channel reduced pancreatitis-associated acute lung injury, suggesting that targeting multiple cell types, including immune cells, is crucial for effectively treating the systemic complications of pancreatitis. This underscores the potential for ORAI1 inhibitors to address both pancreatic and systemic inflammation, such as pancreatitis-associated acute lung injury (206). The function of ORAI1 in SOCE and its subsequent impact on calcium influx has been further elucidated through the application of inhibitors like CM4620, which modulate both parenchymal and immune cell functions. By inhibiting ORAI1, CM4620 reduces acinar cell pathology and inflammatory responses, offering a dual-action approach that targets both tissue damage and immune-mediated inflammation (49). In addition to ORAI1, recent studies have identified the protein SARAF (SOCE-associated regulatory factor) as a protective regulator against excessive Ca^2+^ influx. SARAF degradation during pancreatitis leads to worsened disease severity, highlighting the delicate balance in calcium regulation mechanisms within acinar cells. Both ORAI1 and SARAF are integral to maintaining calcium homeostasis, with SARAF acting to modulate ORAI1 activity and prevent excessive SOCE (207). Moreover, ORAI1’s activity is also regulated by cholesterol, which inhibits its function and limits SOCE. Cholesterol depletion has been found to enhance ORAI1-mediated calcium influx, leading to increased degranulation and inflammatory mediator release. This complex interaction between cholesterol and ORAI1 suggests that lipid regulation may also play a role in modulating the severity of inflammatory responses in conditions like pancreatitis (208).

#### 5.2.4 Energy metabolism regulator

Energy metabolism regulators can enhance the adaptability and antioxidant capacity of pancreatic cells by improving mitochondrial energy metabolism status. Coenzyme Q10, a key player in mitochondrial energy metabolism, has demonstrated a protective effect in treating pancreatitis. Research indicates that coenzyme Q10 can mitigate pancreatic damage and associated pulmonary complications by inhibiting inflammatory cytokines and inflammatory cell infiltration (209). Inorganic phosphate is essential for ATP production, and studies have revealed that phosphate supplementation can prevent experimental pancreatitis by enhancing mitochondrial function (210, 211).

#### 5.2.5 Anti-inflammatory agents

Anti-inflammatory drugs have shown promise in protecting the pancreas by modulating mitochondrial function and reducing oxidative stress. However, the precise mechanisms by which these drugs exert their protective effects, particularly in relation to mitochondrial function, remain a subject of ongoing research and debate.

Some studies indicate that certain anti-inflammatory drugs may have direct effects on mitochondria. For instance, Tanshinone I, a Chinese herbal ingredient known for its anti-inflammatory and antioxidant properties, has been studied for its ability to mitigate mitochondrial damage, oxidative stress, and apoptosis in AP models (212, 213). Moreover, quercetin enhance mitochondrial bioenergetics and protect pancreatic β-cells from cholesterol-induced mitochondrial dysfunction, further mitigating inflammatory responses in pancreatitis (214). Similarly, aspirin, a widely used Nonsteroidal Anti-inflammatory Drug (NSAID), can uncouple oxidative phosphorylation in liver mitochondria, suggesting a direct mitochondrial interaction (215).

The primary mechanism by which anti-inflammatory drugs protect mitochondria in AP is likely indirect, through the reduction of overall inflammation. Inflammatory mediators, particularly cytokines, can significantly impair mitochondrial function. TNFα, a key inflammatory cytokine, has been linked to the clinical prognosis of AP (216). TNFα can directly damage mitochondria in pancreatic acinar cells, leading to ATP depletion and cell death (217). By reducing levels of these inflammatory mediators, anti-inflammatory drugs may indirectly preserve mitochondrial function. For example, TNFα inhibitors like Infliximab have been shown to reduce systemic inflammatory responses and decrease mortality in experimental pancreatitis (218). Some anti-inflammatory drugs have demonstrated specific mechanisms in protecting mitochondria. For instance, pentoxifylline has been shown to protect against mitochondrial damage in experimental pancreatitis by modulating glutathione levels and nitric oxide, showcasing its ability to attenuate inflammatory responses (219).

Anti-inflammatory drugs show promise in protecting pancreatic mitochondria during AP, but their precise mechanisms remain unclear. While some drugs may have direct effects on mitochondrial function, their primary benefit likely stems from reducing overall inflammation, which indirectly supports mitochondrial health. The complexity of AP models makes it challenging to isolate direct mitochondrial effects from broader cellular responses. Future research should focus on specifically measuring mitochondrial function parameters in response to anti-inflammatory treatments, distinguishing between direct and indirect effects, and examining the temporal relationship between mitochondrial changes and inflammatory marker reductions. Understanding these nuanced mechanisms is crucial for developing more targeted and effective therapeutic approaches for AP. Exploring potential synergies between traditional anti-inflammatory drugs and targeted mitochondrial therapies may open new avenues for treatment.

#### 5.2.6 Drugs for mitochondrial function repair

Mitochondrial function repair aims to safeguard pancreatic cells from inflammatory and pathological processes by enhancing mitochondrial function, reducing oxidative stress, and mitigating cell damage. High-temperature requirement protein A2 (HtrA2/Omi) is a crucial mitochondrial protease involved in maintaining mitochondrial proteostasis. Research indicates that deoxyarbutin inhibits oxidative stress, restores impaired mitochondrial function, and ameliorates pancreatic damage through a pathway dependent on HtrA2/PGC-1α (220). Furthermore, diosgenin and its derivative dihydrodiosgenin exhibit protective effects on mitochondria by preventing mitochondrial depolarization, ATP depletion, ROS generation, and excessive inflammatory responses, thereby improving lung injury associated with AP (221).

Mitochondrial drug protection is a promising therapeutic approach for pancreatitis, aimed at safeguarding pancreatic cells from inflammation and damage by modulating mitochondrial function and reducing mitochondrial damage and oxidative stress. Compounds like cyclosporine A, N-acetylcysteine, coenzyme Q10, and tanshinone show potential for mitochondrial protection in pancreatitis. Nevertheless, additional research and validation are needed for the clinical implementation of mitochondrial drug protection in pancreatitis treatment.

#### 5.2.7 Mitophagy regulator

Mitochondrial dysfunction and impaired autophagy are hallmarks of AP, contributing to the pathological damage in pancreatic acinar cells. Enhancing mitophagy has emerged as a promising therapeutic strategy to restore mitochondrial integrity, promote mitochondrial biogenesis, and improve ATP production, all of which are critical for pancreatic cell survival and function in AP.

Specific agents that regulate mitophagy have shown potential in addressing mitochondrial dysfunction in pancreatic tissue. For instance, melatonin, a well-known antioxidant, has demonstrated significant protective effects in various disease models. In polycystic ovary syndrome, melatonin enhances SIRT1 expression, inhibiting excessive PINK1/Parkin-mediated mitophagy and ameliorating mitochondrial dysfunction both *in vitro* and *in vivo* (222). While direct studies in AP are limited, these findings suggest melatonin’s potential in modulating mitochondrial quality control in pancreatic inflammation. In AP specifically, melatonin exhibits protective effects through its potent antioxidant, anti-inflammatory, anti-apoptotic, and anti-hyperlipidemic properties, improving both histological and biochemical parameters (223). Another promising approach involves the inhibition of mitoNEET, a key regulator of mitochondrial function. This inhibition enhances mitophagy by activating PINK1-Parkin signaling, promoting the clearance of dysfunctional mitochondria and maintaining cellular energy balance (224). Recent studies have highlighted the role of mitoNEET in pancreatic cells, offering insights into its potential relevance for AP. While mitoNEET induction in β-cells activates a Parkin-dependent mitophagy pathway that may impair glucose-stimulated insulin secretion, its induction in α-cells promotes anti-apoptotic effects and enhances insulin secretion, indirectly supporting β-cell function (225). These findings underscore the nuanced role of mitoNEET in maintaining pancreatic cell homeostasis and highlight its emerging importance in pancreatic research.

Other mitophagy regulators, such as probucol, offer distinct mechanisms by improving mitochondrial quality without relying on PINK1/Parkin pathways, highlighting its potential in lipid droplet regulation and mitochondrial protection (226). Similarly, isorhamnetin prevents mitochondrial dysfunction by reducing ROS generation, making it a promising candidate for severe AP treatment (227). Additionally, rapamycin, an mTOR inhibitor, enhances mitophagy and alleviates pancreatitis by restoring autophagy flux and reducing endoplasmic reticulum stress (228).

These findings collectively underscore the translational potential of mitophagy regulators from broader research contexts to specific applications in pancreatic inflammation.

## 6 Prospects and challenges of mitochondrial therapy in acute pancreatitis

Mitochondria have traditionally been recognized as key players in regulated cell death, with research indicating that the disruption of mitochondrial function and structure during this process is closely linked to the inflammatory response that helps maintain overall body balance. Dysregulated inflammatory responses triggered by mitochondrial components or products have been implicated in various human diseases. Many of these diseases can be managed clinically through therapeutic approaches that target inflammatory mediators or PPRs and their signaling pathways (229).

Mitochondrial therapy offers promising prospects in treating AP by targeting mitochondrial dysfunction to alleviate inflammation and cellular damage. Innovations such as mitochondria-targeted antioxidants (e.g., MitoQ, Tiron) have shown superior efficacy in neutralizing oxidative stress and protecting mitochondrial integrity. Mitochondrial transplantation has demonstrated therapeutic benefits in various organ injuries, suggesting its potential for restoring mitochondrial function in pancreatitis. Additionally, pharmacological interventions, including Cyclosporin A and energy metabolism regulators like Coenzyme Q10, show potential in preventing mitochondrial dysfunction and reducing cellular damage, making mitochondrial therapy a promising approach for improving outcomes in AP.

Although mitochondrial therapy shows promising potential for treating AP, several challenges need to be addressed, including optimizing delivery methods, maintaining mitochondrial viability during transfer, and evaluating long-term safety and efficacy (230). Moreover, extensive clinical trials are necessary to validate the efficacy and safety of these therapies. Navigating regulatory hurdles is crucial to facilitate their translation into clinical practice. Developing efficient delivery systems for targeted and sustained release of mitochondrial therapeutics poses a significant obstacle. Ensuring precise targeting to affected pancreatic cells without off-target effects is also critical. Furthermore, a comprehensive investigation into the pivotal role of mitochondrial dysfunction in the pathogenesis of pancreatitis and elucidation of the underlying molecular mechanisms are crucial for the diagnosis and treatment of AP.

Future research should focus on refining mitochondrial transplantation techniques, developing targeted delivery systems to enhance uptake by pancreatic cells, and exploring synergies with antioxidant or anti-inflammatory therapies. Additionally, rigorous validation in AP-specific animal models and clinical studies is essential to establish its therapeutic potential. By activating mitophagy, mitigating inflammation, and restoring cellular energetics, this strategy offers a novel paradigm in AP treatment. Continued advancements in mitochondrial transplantation techniques could pave the way for transformative interventions, providing new hope for patients suffering from this severe condition.

## 7 Conclusions

Mitochondria play a crucial role in the pathogenesis of AP, an inflammatory condition affecting pancreatic tissue. The development of AP is intricately linked to mitochondrial dysfunction, resulting in reduced ATP production, oxidative stress, and loss of mitochondrial membrane potential. This dysfunction causes an increase in mitoROS production, exacerbating cellular damage and inflammation. Furthermore, mitochondrial dysfunction can trigger apoptosis and necrosis by releasing proteins like Cytc into the cytoplasm. AP also induces mutations and oxidative damage in mitochondrial DNA, further compromising mitochondrial function and cellular metabolism. Therefore, exploring the involvement of mitochondria in AP is crucial for understanding the disease mechanism and identifying potential therapeutic targets. Future research efforts should focus on developing strategies to repair and protect mitochondrial function in order to enhance the effectiveness of treatments for AP.
